# In vitro interaction of *Metarhizium anisopliae* Ma9236 and *Beauveria bassiana* Bb9205 with *Heterorhabditis bacteriophora* HNI0100 for the control of *Plutella xylostella*

**DOI:** 10.1186/s40064-016-3745-5

**Published:** 2016-12-01

**Authors:** J. P. Correa-Cuadros, A. Sáenz-Aponte, M. X. Rodríguez-Bocanegra

**Affiliations:** 1 Laboratorio de Control Biológico, Grupo de Biología de Plantas y Sistemas Productivos, Departamento de Biología, Facultad de Ciencias, Pontificia Universidad Javeriana, Bogotá, D.C. Colombia; 2 Unidad de Investigaciones Agropecuarias, Departamento de Microbiología, Facultad de Ciencias, Pontificia Universidad Javeriana, Bogotá, D.C. Colombia

**Keywords:** Broccoli, Biological control, Diamondback moth, Entomopathogenic fungi, Entomopathogenic nematodes

## Abstract

The diamondback moth (*Plutella xylostella*) is a major pest of broccoli crops in Colombia. To control *P. xylostella*, we evaluated the interaction of *Beauveria bassiana* Bb9205 and *Metarhizium anisopliae* Ma9236 with *Heterorhabditis bacteriophora* HNI0100 and its bacterial symbiont *Photorhabdus luminescens* HNI0100. We used antagonism and disk diffusion assays with fungal extracts to test the interaction between symbiotic bacterium and fungi. *P. luminescens* inhibited the growth of *B. bassiana* and *M. anisopliae* up to 40% by the secretion of secondary metabolites, whereas fungal extracts did not inhibit *P. luminescens*; this explains the in vivo interactions of these biological control agents. To test the interaction between fungi and nematodes, we first inoculated the fungi followed by the nematodes on different days (0, 2, 4, and 6). We identified the type of interaction using the formula by Nishimatsu and Jackson (J Econ Entomol 91:410–418, 1998) and established that on days 0, 2 and 4 there was an antagonistic interaction, while a synergistic interaction occurred on day 6. Therefore, the use of the interaction between *H. bacteriophora* HNI0100 with *M. anisopliae* Ma9236 and *B. bassiana* Bb9205 is an innovative alternative for the control of *P. xylostella*.

## Background

*Plutella xylostella* (Linnaeus, 1758) (Lepidoptera: Plutellidae), known as the diamondback moth, is one of the major pests of broccoli crops (Bertolaccini et al. 2010; Sáenz 2012). In Colombia and worldwide, the diamondback moth generates annual crop losses greater than 80% (Ochoa et al. 1989; Verkerk and Wright 1996; Sarfraz et al. 2005). The most serious damage is caused by thirdth and fourth instar larvae, which feed on the abaxial surface of leaves (Somvanshi and Ganguly 2007), altering the photosynthetic process and reducing the size and quality of the product for consumption (Chávez and Hurtado 2010; Correa-Cuadros et al. 2014).

Control of this pest is achieved through the constant use of insecticides such as organophosphates, carbamates and pyrethroids (Franco 2001; Furlong et al. 2013). However, their indiscriminate use has increased the pest’s resistance (Monzón 2001; Furlong et al. 2013). For this reason, biological control has been evaluated as an alternative to reduce populations of *P. xylostella* and found that entomopathogenic fungi and nematodes have revealed significant advances (Bertolaccini et al. 2010). The use of fungi in laboratory conditions presents high pest mortality percentages (*Beauveria bassiana* Bb9205, 95.33%, and *Metarhizium anisopliae* Ma9236, 99.67%), but this mortality percentages are achieved in a long period time (9–15 days); while entomopathogenic nematodes, *Heterorhabditis bacteriophora* HNI0100, reach a mortality of 91.66% in 48 h (Correa-Cuadros et al. 2014). Barbercheck and Kaya (1990) established that the use of a combination of biological control agents generates interactions that increase the percentage of pest mortality by 80% in different pests, which demonstrates the efficiency of combinations in the production of epizootics.

Ansari et al. (2004, 2008), Koppenhöfer and Grewal (2005) and Tarasco et al. (2011) cite that combinations between entomopathogenic nematodes and fungi can generate interactions such as synergism that increase pest mortality, antagonism in which a biological control agent inhibits another by competing for space and resources, and additivity when they act independently of each other. To date, these interactions have not been elucidated in *P. xylostella*; therefore determining the relationships between the studied fungal and nematode strains is essential to understand these microorganisms behavior in the host. For that reason, the purpose of this study was to evaluate the interaction of *B. bassiana* Bb9205 and *M. anisopliae* Ma9236 with *H. bacteriophora* HNI0100 to control *P. xylostella*.

## Results

### Antagonism between *B. bassiana* Bb9205 and *M. anisopliae* Ma9236 with *P. luminescens* HNI0100

**Fig. 1:**
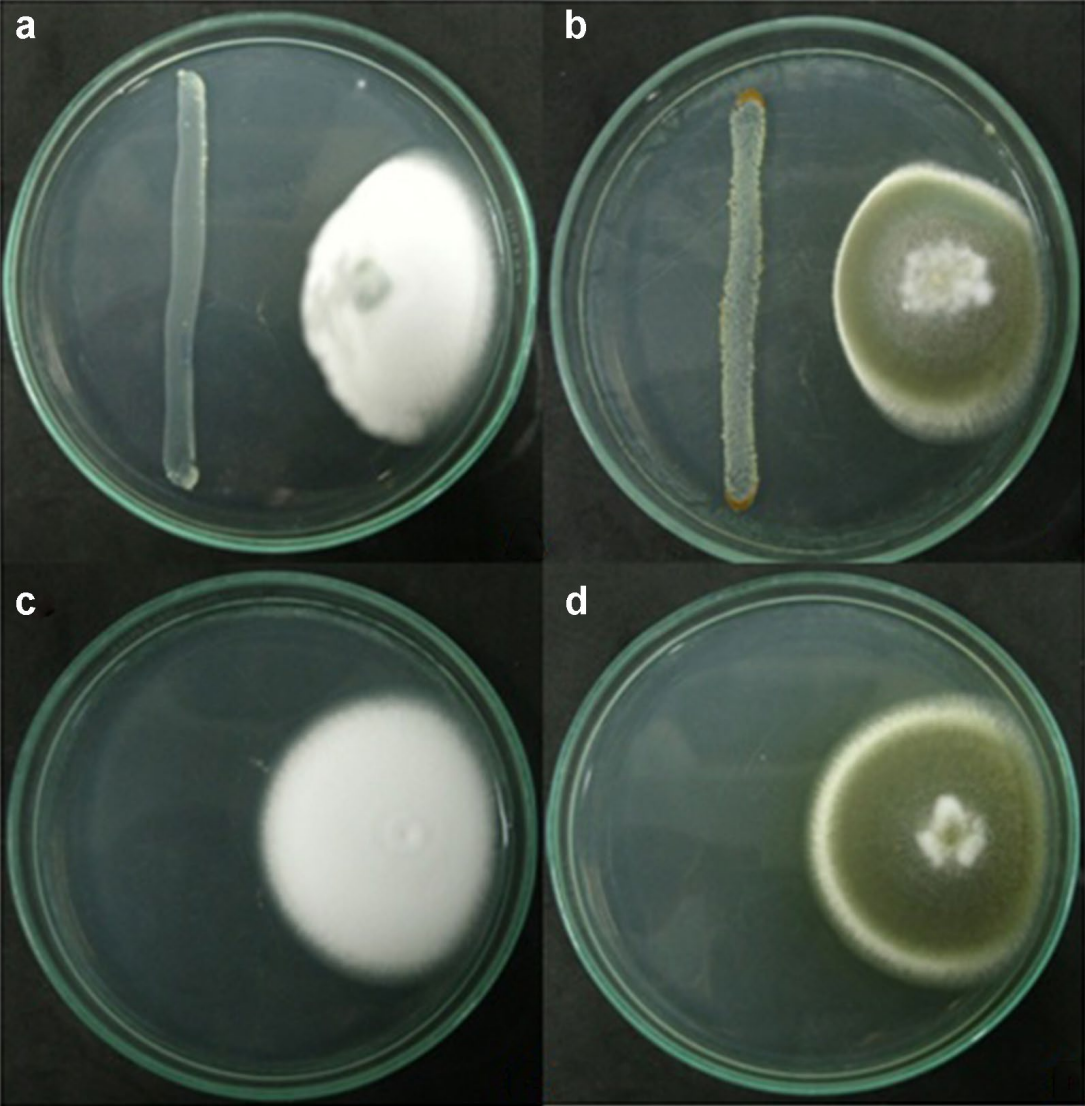
Antagonism test to evaluate the growth inhibition of *B. bassiana* Bb9205 and *M. anisopliae* Ma9236 with *P. luminescens* HNI0100. **a** *P. luminescens* and *B. bassiana.* **b** *P. luminescens* and *M. anisopliae*. **c** *B. bassiana* control. **d** *M. anisopliae* control

The percentage of reduction in fungus radial growth for *B. bassiana* Bb9205 was 48% ± 0,5 and 30% ± 1,8 for *M. anisopliae* Ma9236 (Fig. 1). The secondary metabolites of *P. luminescens* HNI0100 were able to significantly inhibit radial growth of both fungi (*F* = 4.408; *df*_1_ = 18; *df*_2_ = 2; *p* = 0.028). The *P. luminescens* HNI0100 metabolites inhibited *B. bassiana* Bb9205 more than *M. anisopliae* Ma9236 (*p* < 0.05 in the Tukey and Scheffe tests). *P. luminescens* HNI0100 was not inhibited by *B. bassiana* Bb9205 and *M. anisoplia*e Ma9236 fungal extracts, in any of the treatments (10, 100 or 1000 µg crude extract).

### Interaction of *B. bassiana* Bb9205 and *M. anisopliae* Ma9236 with *H. bacteriophora* HNI0100

**Fig. 2:**
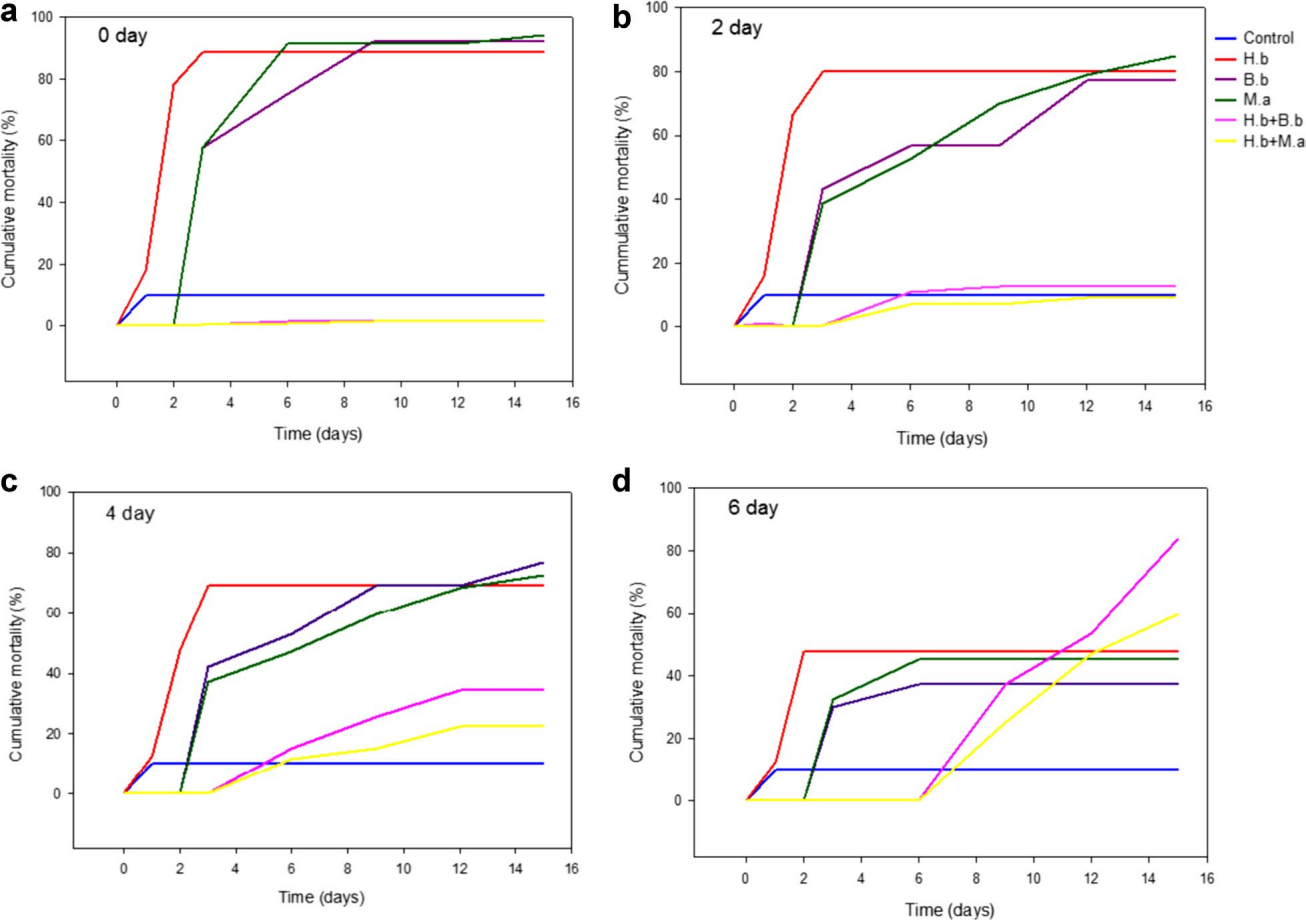
Interaction of *B. bassiana* Bb9205 and *M. anisopliae* Ma9236 with *H. bacteriophora* HNI0100 on *P. xylostella* at different application times of *H. bacteriophora* HNI0100. **a** 0 day. **b** 2 day. **c** 4 day. **d** 6 days. B.b = *B. bassiana* Bb9205, M.a = *M. anisopliae* Ma9236, H.b = *H. bacteriophora* HNI0100

The interaction between the entomopathogenic fungi and nematode was manifested by the high mortality porcentages of *P. xylostella* larvae observed in the treatments which combine the application of both pathogens (6 day) (Fig. 2). The treatments (control, *H. bacteriophora*, *B. bassiana*, *M. anisopliae*, *B. bassiana* + *H. bacteriophora* and *M. anisopliae* + *H. bacteriophora*) showed significant differences (*F* = 3.151; *df*_1_ = 118; *df*_2_ = 5; *p* = 0.0001); *H. bacteriophora* HNI0100 had the highest mortality (70%) compared to fungi and nematode combinations and individually applied fungi, independent of application time.

We found significant differences (*F* = 3.229; *df*_1_ = 118; *df*_2_ = 3; *p* = 0.0001), regarding application times (0, 2, 4 and 6 days). The *H. bacteriophora* tests carried out on days 0, 2, 4 and 6 achieved a mortality rate of 70%. For *B. bassiana*, from 0 to 2 days mortality was 60% and from 4 to 6 days, 68%. For the four application times, *M. anisopliae* obtained 58% mortality.

In comparison to the results of individually applied biological control agents, combination treatments presented marked differences depending on application time. For 0 and 2 days, the combined infection between fungi and nematode presented a low mortality percentage (2–15%). On day 4, mortality increased by 25–35%. However, day 6 revealed a significant difference; the percentages exceeded those of individually applied control agents, 85% for *B. bassiana* + *H. bacteriophora*, and 60% for *M. anisopliae* + *H. bacteriophora*. This confirms that the highest mortality in *P. xylostella* is obtained by first applying *B. bassiana* Bb9205 and *H. bacteriophora* HNI0100 6 days later.

**Fig. 3:**
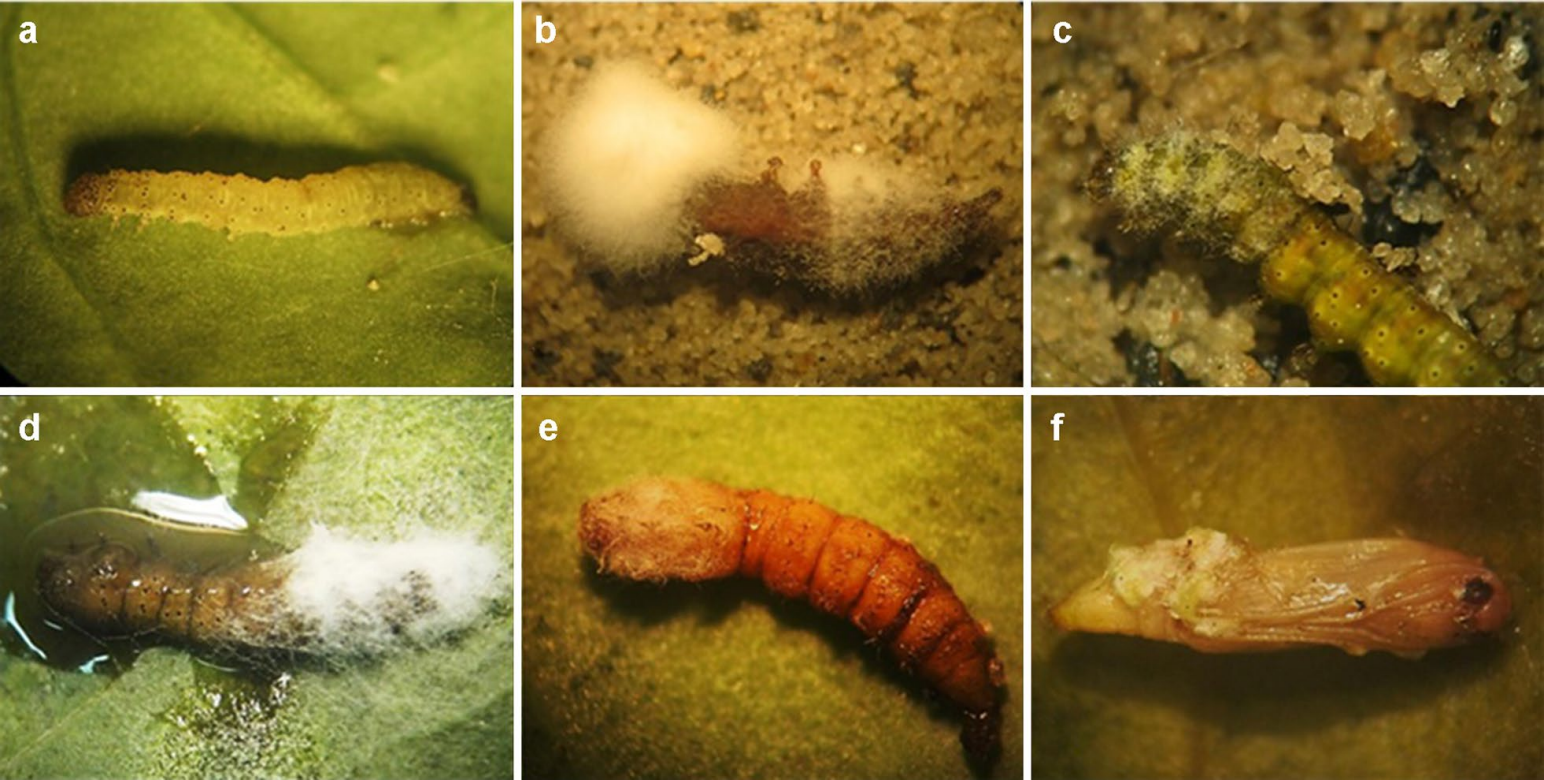
Interaction of *B. bassiana* Bb9205 and *M. anisopliae* Ma9236 with *H. bacteriophora* HNI0100 on *P. xylostella*. **a** *P. xylostella* control. **b** Larva infected by *B. bassiana* Bb9205 and *H. bacteriophora* HNI0100 (day 6). **c** Larva infected by *M. anisopliae* Ma9236 and *H. bacteriophora* HNI0100 (day 6). **d** Larva infected by *B. bassiana* Bb9205 and *H. bacteriophora* HNI0100 (day 4). **e** Interaction of *M. anisopliae* Ma9236 with *H. bacteriophora* HNI0100 (day 4). **f** Pupa infected by *B. bassiana* Bb9205 and *H. bacteriophora* Ma9236 (day 6)

The interactions observed in χ^2^ (Table 1) show that day 0, 2 and 4 tests had an antagonistic interaction as the values were lower than those expected (Fig. 3). Conversely, the values on day 6 were higher than expected, which determine a synergistic interaction (Fig. 3). The mortality percentages observed in the dual infections exceed the sum of the individual effects, this demonstrates the existing synergy.

**Table 1:** Interaction of *B. bassiana* Bb9205 and *M. anisopliae* Ma9236 with *H. bacteriophora* HNI0100 on *P. xylostella*, at different application times of *H. bacteriophora* HNI0100 (0, 2, 4 and 6 days)

Test^a^	Fungus^b^	Observed mortality^c^	Expected mortality^d^	χ^2^	Interaction^e^
Day 0	B.b	3	45.3	40.4	Antagonism
Day 0	M.a	2	42.5	38.6	Antagonism
Day 2	B.b	15	44.3	24.35	Antagonism
Day 2	M.a	10	41.5	22.33	Antagonism
Day 4	B.b	35	46.3	4.65	Antagonism
Day 4	M.a	25	45.5	5.58	Antagonism
Day 6	B.b	85	50.3	12.12	Synergy
Day 6	M.a	70	47.5	9.92	Synergy

^a^Interval between application of entomopathogenic fungi and *Heterorhabditis bacteriophora*

^b^B.b, *B. bassiana*; M.a, *M. anisopliae*

^c^Mortality observed for the combination of the two control agents

^d^Expected mortality (%) = P_0_ + (1 − P_0_) (P_1_) + (1 − P_0_) (1 − P_1_) (P_2_), where P_1_ is mortality from nematodes alone, P_2_ is mortality from other

^e^Interaction based on the expected χ^2^ and the observed mortality

## Discussion

### Antagonism between *B. bassiana* Bb9205 and *M. anisopliae* Ma9236 with *P. luminescens* HNI0100

The antagonistic interaction of *B. bassiana* Bb9205 and *M. anisopliae* Ma9236 with *P. luminescens* HNI0100 may be due to the production of antifungal substances (Forst and Clarke 2002; Webster et al. 2002; Waterfield et al. 2009) that inhibit conidial germination and the elongation of the germinal tube of some fungi (Chen et al. 1996; Ffrench-Constant et al. 2007); in other cases, by a decrease in the rate of reproduction and germination (Barbercheck and Kaya 1990; Ng and Webster 1997; Silva et al. 2002). Chen et al. (1996) and Ansari et al. (2004) have cited that when bacteria produce their virulence factors they influence the behavior of the accompanying microorganisms, altering their growth and reproduction. One example is the study by Molina et al. (2007) in which *P. luminescens* inhibited the growth of a wide range of entomopathogenic fungi when it produced antimicrobials for 21 days in the host.

Among the possible compounds produced by *P. luminescens* HNI0100 are stilbenes; these compounds alter hormonal balance and interrupt physiological processes (Sánchez 2002; Waterfield et al. 2009) and are active against a broad range of microbial species such as fungi. Richardson et al. (1988) and Hu and Webster (2000) reported a *Photorhabdus* strain that produced higher levels of 3,5-dihydroxy-4-ethyl stilbene during the post-exponential phase in *G. mellonella* larvae, and were active against the intestinal bacteria of the host. Additionally, there are other complex compounds that counteract fungal growth, such as polyketides with antimicrobial activity, toxins Tca, Tcb, Tcc, Tcd, Mcf1, Mcf2, PirAB, S and R-type pyocins, PVC and anthraquinones (Ffrench-Constant et al. 2007; Waterfield et al. 2009; Nielsen-Leroux et al. 2012).

Besides the toxins, enzyme complexes such as lipases, proteases, lecithinases and phospholipases also promote septicemia in the insect and antagonistic microorganisms in the host (Sánchez 2002). It is possible that the bacteria’s primary and secondary metabolism secreted enzymes such as proteases (alkaline metalloproteases) and chitinases that degrade tissues and inactivate the defense system of fungi (Chen et al. 1996; Forst and Clarke 2002). Similarly, lipases break lipid chains (Silva et al. 2002; Forst and Clarke 2002), lecithinases decompose phospholipids and destroy cells obstructing fungal growth (Bowen et al. 1998). Furthermore, endo and exochitinases which degrade fungal cell walls, may inhibit fungal growth and reproduction as evidenced in *B. bassiana* Bb9205 and *M. anisopliae* Ma9236 (Chen et al. 1996; Ng and Webster 1997).

The growth inhibition percentage of *B. bassiana* Bb9205 and *M. anisopliae* Ma9236 was 48 and 30%, respectively; these are considered low when compared to percentages obtained by Tarasco et al. (2011) who suggest that inhibition ranges exceeding 60% are caused by highly virulent strains. Ansari et al. (2005) performed antagonism tests, where *P. luminescens* were antagonistic to *M. anisopliae*, *B. bassiana*, *B. brongniartii*, and *Paecilomyces fumosoroseus* by inhibiting the growth and production of conidia. In this study, there were established percentages of 40% inhibition for *B. bassiana* and 33% for *M. anisopliae*; the results were similar to those obtained in this study. This inhibition is based on antagonistic interaction mechanisms between microorganisms; this may be based on parasitism, direct competition and antibiosis.

The diffusion test of crude extracts of *M. anisopliae* Ma9236 and *B. bassiana* Bb9205 with *P. luminescens* HNI0100 evidenced that there is no inhibition at the concentrations used (10, 100 and 1000 µg). This confirms that Ma9236 and Bb9205 strains have no antimicrobial or antagonistic activity; contrary to studies such as Ansari et al. (2005) in which extracts of *M. anisopliae* showed antagonistic activity against *P. luminescens*, proving their antimicrobial abilities at concentrations of 1000 µg/mL.

### Interaction of *B. bassiana* Bb9205 and *M. anisopliae* Ma9236 with *H. bacteriophora* HNI0100

The interaction between fungi and nematodes can be additive, synergistic or antagonistic depending on the control species, the insect pest, and infection dose and application time, according to Ansari et al. (2008, 2010). An additive effect is determined when two control agents act independently from each other in a combined infection, whereas in a synergistic or antagonistic interaction, the interaction makes the combination more or less effective than the additive effect (Koppenhöfer and Grewal 2005). Ansari et al. (2010) and Koppenhöfer and Grewal (2005) suggest that when the nematodes and fungi are inoculated at the same time, their interaction have an additive effect on host mortality, as the two control agents act independently. In this study, however, these effects were not present in any of the application times (0, 2, 4 and 6 days). According to Ansari et al. (2010), although additive effects occur in most dual infections, in some cases, antagonistic interactions may occur. On day 0, 2 and 4, of this study, mortality of the individually applied biological control agents was higher than in the dual infections; this being the case, antagonism in the interaction of both controllers is confirmed since the percentage was less effective than the sum of each individually applied. The symptomatology of the larvae was characteristic for entomopathogenic nematodes, and there was no presence of mycelium.

Antagonistic interactions were also present in studies by Tarasco et al. (2011), competition for survival space in the host’s hemocoel and food resources was evident revealing that *Xenorhabdus bovienii* and *B. bassiana* are antagonistic; this interaction occurs because the nematode is considered more virulent. The nematode achieves faster colonization because of its active dispersion; it enters the larvae within 24 h, whereas the fungi take 2–3 days. It should be noted that, once the fungus penetrates the host, the bacterial symbiont is in its exponential phase producing primary and secondary metabolites with antimicrobial activity (Molina et al. 2007; Tarasco et al. 2011). Unlike the data obtained here, Molina et al. (2007) and Ansari et al. (2008) show that it is possible to establish positive interactions, possibly synergistic or additive, by applying entomopathogenic fungi and nematodes such as *M. anisopliae* and *H. bacteriophora* at the same time or in short intervals of 2 days, with other pests.

On the time 6 day, we obtained the only synergistic interaction presenting a high mortality rate of *P. xylostella* larvae caused by the combined infection (*H. bacteriophora* HNI0100 with *M. anisopliae* Ma9236 and *B. bassiana* Bb9205); this exceeds the sum of the independent effects. This interaction has been confirmed in simultaneous applications of different control agents such as *B. brongnartii*, and *Heterorhabditis* sp. on *Otiorrhynchus sulcatus* larvae (Sánchez 2002) or on *Hoplia philanthus* larvae by infections of *M. anisopliae* and *H. bacteriophora* (Ansari et al. 2004). This establishes that, for a synergistic interaction to occur time is key to allow the life cycles of both controllers and although competition for space and nutrients may occur, they do not completely inhibit each other (Ansari et al. 2006). Ansari et al. (2004, 2008) suggest that one of the main factors of a synergistic interaction is the weakening of larvae by an initial fungal infection, which inhibits the larvae from feeding normally. The fungus also increases the host’s susceptibility to nematodes by generating a stressful condition and altered behavior (Ansari et al. 2006, 2008). In addition, larvae emit a higher concentration of CO_2_ in response to fungal colonization, which makes these emissions reach nematodes quickly and efficiently locate those (Ansari et al. 2004). The data obtained confirm that the degree of interaction varies in time. Ansari et al. (2004, 2008) suggest that, in positive interactions, nematodes must be added after the fungi. These intervals should be determined according to the host as production of antimicrobials by symbiotic bacteria which can affect the fungi, due to the slower development and secondary metabolites production of fungi (Tarasco et al. 2011).

In conclusion, the combined use and interactions of *H. bacteriophora* HNI010 with *M. anisopliae* Ma9236 and *B. bassiana* Bb9205, can be considered as an innovative alternative for the control of *P. xylostella* and could be considered as a management strategy in broccoli crops.

## Methods

### Studied microorganisms

For laboratory testing, entomopathogenic fungi *B. bassiana* Bb9205, and *M. anisopliae* sensu lato Ma9236 were used. These isolates were obtained from the Laboratorio de Control de Calidad de Bioinsumos Agrícolas—Control de Bioinsumos Disciplina de Entomología, Cenicafé, Chinchina-Caldas—Colombia. The pure strains were plated in PDA (potato dextrose agar) and Oatmeal Agar for 15 days at 25 °C, to achieve optimal growth and conidia production. Following the methodology of Kaya and Stock (1997), we multiplied the entomopathogenic nematode *H. bacteriophora* HNI0100, supplied by the Biological Control Laboratory at the Pontificia Universidad Javeriana (Delgado-Ochica and Sáenz 2012), in vivo by infection of last instar larvae of *Galleria mellonella* (Linnaeus, 1756) (Lepidoptera: Pyralidae).

*Photorhabdus luminescens* HNI0100 was isolated from last instar larvae of *G. mellonella* infected with infective juveniles of *H. bateriophora* HNI0100. To isolate the bacterial symbiont, the second intersegmental membrane of the larvae was punctured to extract the hemolymph, which was plated on NBTA for 48 h (Chen et al. 1996), to validate bacterial morphology and bioluminescence (Saénz-Aponte et al. 2014).

### Antagonism between *B. bassiana* Bb9205 and *M. anisopliae* Ma9236 with *P. luminescens* HNI0100

To evaluate fungal growth inhibition against the symbiotic bacterium, antagonism tests were performed between *B. bassiana* Bb9205 and *M. anisopliae* Ma9236 with *P. luminescens* HNI0100. To do this, 40 µL of a 1:1 suspension of water agar and 1 × 10^8^ conidia/mL of each fungus were inoculated in a well bored at 3 cm from the edge of a PDA plate. The bacterial inoculum was adjusted to 0.2 McFarland scale and streaked 3 cm from the edge of the petri dish, in front of the fungus. Each treatment had five replicates and three time repetitions. The fungal growth inhibition percentage was monitored for 2 weeks at 25 °C.

A disk diffusion assay was performed to evaluate the growth inhibition of *P. luminescens* HNI0100 by the action of fungal metabolites. To this end, fungal secondary metabolites were extracted from 10 days cultures on Czapek broth supplemented with peptone (fungal inoculum 1 × 10^8^ conidia/mL). The cell free culture broth, obtained by centrifugation at 4000 rpm for 15 min, was extracted with dichloromethane (liquid–liquid extraction ratio 1:1), then the solvent fraction was concentrated to complete dryness in a Buchi (B-490) rotaevaporator at 40 °C and 50 rpm. The filter paper disks were activated with 10, 100 and 1000 µg of extract by adding 10 µL of 1, 10 and 100 mg/mL extracts, respectively. The dried extracts were dissolved in dimethyl sulfoxide (DMSO 20% v/v) to activate the filter paper disks. The agar plates were inoculated with 0.1 mL of *P. luminescens* cells suspension (1 × 10^6^ CFU/mL) (Tarasco et al. 2011). Five replicates and three repetitions at different times were performed.

### Interaction of *B. bassiana* Bb9205 and *M. anisopliae* Ma9236 with *H. bacteriophora* HNI0100

To identify the interactions between the entomopathogenic nematode and both fungi, concentrations of 1 × 10^5^ conidia/cm^2^ of *B. bassiana* Bb9205 and *M. anisopliae* Ma9236 and 1 × 10^2^ JIs/cm^2^ for *H. bacteriophora* HNI0100 were used. The experimental units consisted of a third instar larvae of *P. xylostella* with 60 g of sterile river sand and 5 g of broccoli leaf contained in a 180 mL vessel (therefore the river sand covered a surface area of 33.2 cm^2^ and a depth of 2.5 cm). The interaction experiment comprised 20 experimental units per treatment and three repetitions at different times (Table 2). The entomopathogens were applied over the experimental unit surface.

**Table 2:** Treatments used to evidence the interaction of *Beauveria bassiana* Bb9205 and *Metarhizium anisopliae* Ma9236 with *Heterorhabditis bacteriophora* HNI0100

Test	Treatment 1	Treatment 2	Treatment 3	Treatment 4	Treatment 5	Treatment 6
Day 0	B.b + H.b + P.x	M.a + H.b + P.x	B.b + P.x	M.a + P.x	H.b + P.x	P.x
Day 2	B.b + H.b + P.x	M.a + H.b + P.x	B.b + P.x	M.a + P.X	H.b + P.x	P.x
Day 4	B.b + H.b + P.x	M.a + H.b + P.x	B.b + P.x	M.a + P.x	H.b + P.x	P.x
Day 6	B.b + H.b + P.x	M.a + H.b + P.x	B.b + P.x	M.a + P.x	H.b + P.x	P.x

B.b = *B. bassiana* Bb9205, M.a = *M. anisopliae* Ma9236, H.b = *H. bacteriophora* HNI0100 and P.x = *P. xylostella*

To determine the interaction type between biological control agents towards a pest, the pest must be challenged by the microorganisms at different times (Ansari et al. 2008). Four application times, 0, 2, 4 and 6 days were evaluated. In the first test, fungi and nematodes were applied at the same time (0); in the second test, fungi were applied first and nematodes 2 days later; in the third test, fungi were applied first and nematodes 4 days later; and in the fourth test, fungi were applied first, then nematodes 6 days later. The nematode and fungal inocula were sprayed on sand and broccoli leaf. The mortality was assessed during 15 days. The cadavers were transferred to empty plastic containers to monitor fungal or nematode symptomatology.

To assess the type of interaction (additivity, synergism or antagonism), the analysis by Nishimatsu and Jackson (1998) that uses the percentage of expected and observed host mortality was employed. Expected mortality is based on the formula P_E_ = P_0_ + (1 − P_0_) (P_1_) + (1 − P_0_) (1 − P_1_) (P_2_), where P_E_ is the expected mortality of the combination of both pathogens, P_0_ is the control mortality, P_1_ is the mortality of an individually applied pathogen and P_2_ is the mortality of the other individual pathogen. Then χ^2^ test was performed for the observed and expected results, χ^2^ = (L_0_ − L_E_)^2^/L_E_ + (D_0_ − D_E_)^2^/D_E_, where L_0_ is the number of live larvae observed, L_E_ is the expected number of live larvae, D_0_ is the number of dead larvae observed, and D_E_ is the number of expected dead larvae. The interactions are additive if χ^2^ < 3.84, antagonistic if χ^2^ > 3.84 and P_C_ < P_E_, or synergistic if χ^2^ > 3.84 and P_C_ > P_E_, where P_C_ is the observed mortality of the combination and P_E_ is the expected mortality of the combination. According to Shapiro-Ilan et al. (2004), to calculate the expected mortality, P_1_ is mortality from nematodes alone and P_2_ is mortality from fungi alone.

### Statistical analysis

#### Antagonism between *B. bassiana* Bb9205 and *M. anisopliae* Ma9236 with *P. luminescens* HNI0100

The inhibition percentages obtained in both antagonism tests were subjected to variance homogeneity (Levene) and normality (Kolmogorov–Smirnov) tests, where the assumptions of parametric data were met. Then, a completely randomized design, using an ANOVA, was performed to establish significant differences between treatments. Subsequently, multiple comparisons using Tukey and Scheffe tests were performed, under a 95% probability, to identify the treatment with the highest percentage of inhibition. The tests were carried out using SPSS 19 software IBM SPSS Statistics 19 (SPSS, Inc., Chicago, IL, USA).

#### Interaction of *B. bassiana* Bb9205 and *M. anisopliae* Ma9236 with *H. bacteriophora* HNI0100

A completely randomized design with a factorial arrangement using ANOVA was performed to establish significant differences between treatments (Table 2) and tests (0, 2, 4 and 6 days). The individual and combined mortality data were tested for variance homogeneity (Levene) and normality (Kolmogorov–Smirnov); these yielded the expected parametric data. Subsequently, to identify the treatment and test with the highest percentage of inhibition, multiple comparisons using Tukey and Scheffe tests, under a 95% probability, were carried out. The tests were performed using SPSS 19 software.
